# Modelling the Distribution of Forest-Dependent Species in Human-Dominated Landscapes: Patterns for the Pine Marten in Intensively Cultivated Lowlands

**DOI:** 10.1371/journal.pone.0158203

**Published:** 2016-07-01

**Authors:** Alessandro Balestrieri, Giuseppe Bogliani, Giovanni Boano, Aritz Ruiz-González, Nicola Saino, Stefano Costa, Pietro Milanesi

**Affiliations:** 1 Department of Biosciences, University of Milan, Milan, Italy; 2 Department of Earth and Environmental Sciences, University of Pavia, Pavia, Italy; 3 Natural History Museum of Carmagnola, Carmagnola (TO), Italy; 4 Department of Zoology and Animal Cell Biology, University of the Basque Country (UPV/EHU), Vitoria-Gasteiz, Spain; 5 Systematics, Biogeography and Population Dynamics Research Group, Lascaray Research Center, University of the Basque Country, UPV/EHU, Vitoria-Gasteiz, Spain; 6 Independent Researcher, via Quintino Sella 16, Cossato (BI), Italy; Università degli Studi di Napoli Federico II, ITALY

## Abstract

In recent years, the “forest-specialist” pine marten *Martes martes* has been reported to also occur also in largely fragmented, lowland landscapes of north-western Italy. The colonization of such an apparently unsuitable area provided the opportunity for investigating pine marten ecological requirements and predicting its potential south- and eastwards expansion. We collected available pine marten occurrence data in the flood plain of the River Po (N Italy) and relate them to 11 environmental variables by developing nine Species Distribution Models. To account for inter-model variability we used average ensemble predictions (EP). EP predicted a total of 482 suitable patches (8.31% of the total study area) for the pine marten. The main factors driving pine marten occurrence in the western River Po plain were the distance from watercourses and the distance from woods. EP suggested that the pine marten may further expand in the western lowland, whilst the negligible residual wood cover of large areas in the central and eastern plain makes the habitat unsuitable for the pine marten, except for some riparian corridors and the pine wood patches bordering the Adriatic coast. Based on our results, conservation strategies should seek to preserve remnant forest patches and enhance the functional connectivity provided by riparian corridors.

## Introduction

As already noted by MacArthur in the early 1970s, distribution ranges are dynamic and their boundaries can fluctuate greatly as a consequence of both dispersal and temporal variation in environmental conditions [1]. Range fluctuations generally occur over historical time periods, but they have recently been recorded to occur over short time scales due to introductions and environmental change [2, 3].

Climate warming and human-driven habitat modifications are considered the main determinant of range variation, but non-climatic factors, including ecological interspecific interactions [4, 5] and evolutionary dynamics [6, 7, 8] can also drive the magnitude, direction and pattern of range shifts. While recent studies on species’ distributions have mostly focused on the effects of anthropogenic climate change, demonstrating trends at an Earth-scale, information on environmental determinants of range variation at the fine scale of a species’ range is still scarce [9].

Biological invasions, resulting in large-scale, unintentional field manipulations, offer the opportunity to test the effects of environmental factors on the range limits of alien species [10, 11, 12]: the rate of range expansion has been shown to depend on both the life history of invading species and the properties of the landscape invaded [13]. Less attention has been devoted to range fluctuations in autochthonous species, although the (re-) colonization of previously unoccupied areas may provide an invaluable opportunity for investigating the environmental factors that shape species’ ranges [14].

In recent years, the “forest-specialist” pine marten *Martes martes* has been reported to also occur in largely fragmented landscapes of the western part of its European range [15]. In north-western Italy, a recent genetic survey has showed that the pine marten is spreading in intensively cultivated areas north of the River Po, where woods mainly consist of small residual patches or cover the banks of main rivers [16]. While agro-forest systems have been reported to sustain high food availability levels for carnivores [17], in agricultural landscapes dominated by arable land the loss and fragmentation of natural vegetation have been related to a general decline in prey biomass [18], and the trend of land-use change in the Po plain indicates that soil depletion due to urbanisation is still increasing [19].

Pine marten occurrence in such an apparently unsuitable area is likely the result of recent expansion from the Alps [20], where pine marten populations may have benefited by the increase in forest cover over the last decades [16]. Accordingly, and notwithstanding long-term monitoring, no evidence of pine marten occurrence is available for the lowlands of NE Italy [21] and the central Po plain (1980–2000) [22], while a few, recent records have been reported for the lower course of the River Oglio (Lombardy region) [23].

The colonization of the western Po plain by the pine marten has occurred exponentially [24, 25], as is typical for species that disperse over large distances relative to home range size [26].

Besides dispersal abilities, the rate and direction of species’ expansion are influenced by a wide range of factors, including landscape connectivity, the distribution and abundance of suitable habitats and variation in climate and resource availability [27, 28, 29].

Species Distribution Models (SDMs) [30] prove very useful for understanding how landscape influences the spread of colonizing species and predict habitat suitability in neighbouring geographical areas which may be occupied in the near future (*i*.*e*. the potential geographic distribution) [31, 4].

In the last two decades, the development of several modelling techniques has promoted the use of SDMs in several branches of life sciences [32]. As different techniques can yield partially discordant results [33, 34], model evaluation is needed to assess the accuracy of predictions across modelling techniques and either select the model which “best” fits the data or derive ensemble predictions to avoid single model uncertainty [35].

Our aims were (i) to identify the main environmental predictors related to pine marten presence in the western River Po plain and then (ii) predict the potential for its south- and eastwards expansion.

To reach these aims, we collected available occurrence data of the pine marten in the study area and related them to a set of environmental variables by developing nine different SDMs. To account for inter-model variability, we used average ensemble predictions. Ensemble forecasting is particularly useful in modelling expanding species, which may not yet have spread to all suitable habitats, making species-environment relationships difficult to assess [36].

We hypothesised that the probability of pine marten occurrence would depend on both the distribution of wood patches, as the main limiting factor, and distance from major rivers, which may play a role as natural corridors for expansion [37].

## Methods

### Study area

The Po-Venetian alluvial plain (< 300 m above sea level) is the largest in Italy (*ca*. 46,000 km^2^). The pedogenetic and micro-morphological characteristics of the soils of the lower plain, crossed by the River Po (652 km), support high levels of agricultural productivity and are intensively managed for cattle husbandry and modern-industry-based agriculture. Since the second half of the 19^th^ century, widespread urbanisation and industrialisation have led to a progressive depletion of soil resources; built-up areas have progressively increased since the second half of the 20^th^ century, indicating the parallel increase of human presence [38], and currently cover ca. 9% of the area [19]. About 70% of residual forests (*ca*. 2,400 km^2^) are in the western and central plain [39], and either consist of small fragments (mean patch size = 4.5 ha) [40] scattered within the agricultural matrix or, as in most European lowlands [41], cover the banks of major rivers. Climate is sub-continental temperate, with a mean yearly temperature of 12.0°C and mean yearly rainfall of 1000 mm.

### Data collection

**Fig 1:**
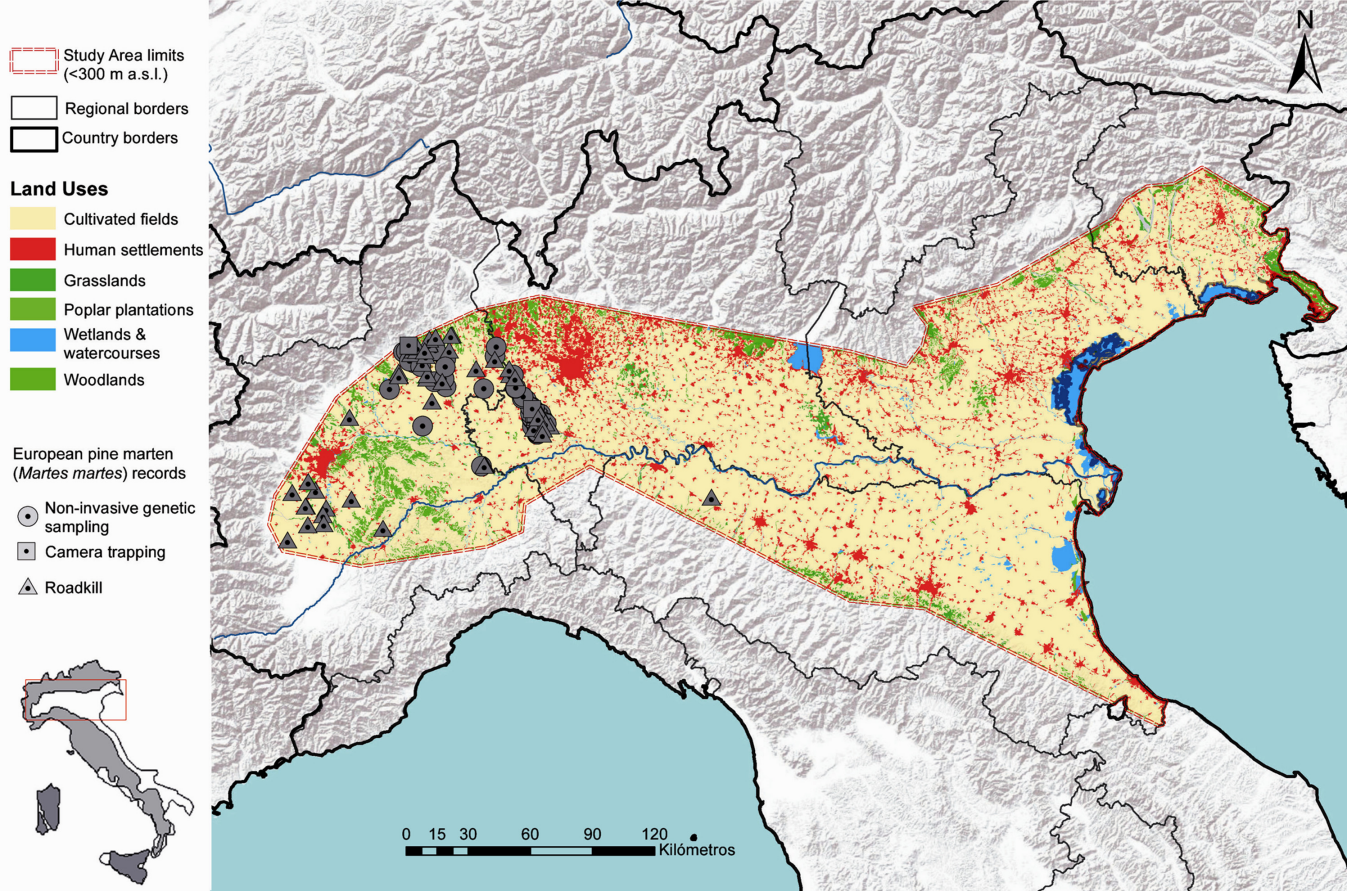
Map of the study area (i.e. the Po-Venetian alluvial plain, < 300 m above sea level) corresponding to the potential expansion range of the pine marten in northern Italy. Pine marten locations are denoted by black triangles (N = 103). Black lines indicate regional borders. The distribution range of the pine marten in Italy is shown in the upper-right corner. Base Map used: World Terrain Base; data sources: Esri, USGS, NOAA; Reprinted from PLOS ONE under a CC BY license, with permission from ESRI, original copyright June 2009.

We collected a total of 184 occurrences for the pine marten recorded between 2000 and 2015 in the Po plain (Fig 1), i.e. available data for north-western Italy were considered below the 300 m a.s.l. contour line, which broadly marks the upper limit of the plain [24]. Two main sources of data were used to assess pine marten distribution: 1) 116 faecal DNA-based records collected between 2007 and 2015 across the whole study area [16, 37], and 2) unequivocal records from road-killed (N = 55) and camera-trapped individuals (N = 13) [42, 25]. All species locations were georeferenced in the UTM WGS84 32N coordinate system using ARCGIS 10.1 (ESRI, Redlands, California, www.esri.com/software/arcgis; S1 Table).

Through Gaussian kernel density estimation based on all sampling locations we accounted for spatially biased sampling effort [43, 44, 45]. Specifically, we used the resulting kernel density probability as a sampling effort map to weight bias-adjusted model estimates [45, 46]. Thus, 10,000 random points within the resulting 95% kernel density surface were generated to serve as background data.

Moreover, according to Calenge et al [47], we estimated the minimum convex polygon (MCP) around all species’ locations to define the area available for the pine marten and to avoid any potential source of bias in the analysis [48].

### Predictor Variables

We collected data on the environmental and anthropogenic features in the whole study area (Table 1). Land cover features, as well as the distance from watercourses and wood patches were derived by the Coordination of Information on the Environment (CORINE Land Cover 2006; http://www.eea.europa.eu/data-and-maps/data/clc-2006-vector-data-version-3), the European land cover database. We measured the land cover percentage of five major habitats: woodland, poplar plantations, grassland, cultivated fields and human settlements. Commercial plantations were distinguished from the woodland category, because they often lack the shrub layer and offer lower prey diversity [49]. We considered these landscape features because of their proven relevance for the distribution of our target species [15, 37] and because they are the most representative in our study area (ca. 98% of the total area). Habitat diversity was expressed by Shannon’s Index. The presence and distance from human settlements (i.e. urban areas, villages; CORINE Land Cover 2006), from roads and railways (derived from OpenStreetMap; www.openstreetmap.org) and human population density (GEOSTAT 2011 dataset—Eurostat—European Commission; ec.europa.eu/eurostat/web/gisco/ geodata/reference-data/ population-distribution-demography) were considered as a proxy for human disturbance.

**Table 1:** Variables used in the development of species distribution models for the pine marten in the whole study area and in the used cells; average ± standard deviations values and variance inflation factor (VIF) values are shown [H’ = − Σ(p_i_ × lnp_i_)].

Variables	Unit	Study area	Used cell	VIF
Cultivated fields	%	70.35 ± 36.37	34.59 ± 33.48	2.540
Grassland	%	0.81 ± 6.18	2.21 ± 8.74	1.086
Poplar plantations	%	2.47 ± 15.47	2.39 ± 13.45	1.065
Woodland	%	4.94 ± 16.74	40.76 ± 33.62	1.688
Habitat Diversity	H’	1.06 ± 0.43	1.48 ± 0.45	1.892
Distance to watercourses	m	5755.01 ± 4583.27	2189.07 ± 2528.82	1.068
Distance to woods	m	8645.01 ± 7511.79	1493.71 ± 2871.78	1.316
Human settlements	%	10.76 ± 21.72	2.17 ± 8.62	2.913
Distance to roads	m	926.11 ± 1242.26	1148.04 ± 977.08	1.284
Distance to human settlements	m	1287.11 ± 1149.11	1980.43 ± 954.02	1.622
Human population density	N/km^2^	388.81 ± 1107.11	41.31 ± 116.61	1.922

All the predictor variables were re-sampled to a common resolution of 1 x 1 km cell size and the variance inflation factor (VIF) for all predictor variables was estimated in order to avoid multi-collinearity among them [50]; VIF values > 3 denoted highly correlated predictors (Table 1).

### Modelling methods

We tested for spatial autocorrelation among all pine marten locations collected with Moran’s *I* correlogram [51] and then, with the resulting non-autocorrelated locations we developed nine SDMs: (1) artificial neural networks (ANN) [52], a non-linear regression model based on hidden variables (estimated by the predictors), (2) boosted regression trees (BRT) [53], a regression model that combines boost methods and regression trees, (3) classification tree analyses (CTA) [54], a recursive partitioning analysis which develops decision trees by applying splitting rules and partitioning data to reduce variation in the response variable, (4) flexible discriminant analyses (FDA) [55], a mixture model-based discriminant analysis, (5) generalized additive models (GAM) [56], a regression model involving smoothing parameters derived by predictor variables to estimate parametric components of linear predictors, (6) generalised linear models (GLM) [57], logistic regression models that relate presence locations and pseudo-absences to the predictors, (7) maximum entropy (MAXENT) [31], a model which calculates a range of functions to identify the best approximation between the density distributions of predictors at species’ occurrences and those in the rest of the study area, (8) multivariate adaptive regression splines (MARS) [58], a non-linear regression which carries out non-linear interactions between variables, (9) random forest (RF) [59], an ensemble classifier involving many decision trees which constitute “the forest”. We used the values of the sampling effort map as a bias grid in MAXENT and as case weights in all the other methods [43, 45, 46]. To avoid single model uncertainty, we calculated the ensemble prediction (EP) derived by the average predictions of the nine SDMs. We converted the EP continuous map into a binary one (suitable/unsuitable) considering a threshold value estimated by maximizing the True Skill Statistics (TSS) [60, 61]; values higher and lower than the threshold represent suitable and unsuitable areas, respectively. To estimate variable importance, we used 10,000 permutations (values close to 0 assume no influence of that variable on the model) [32]. We computed these analyses with the package BIOMOD2 [61] in the open-source software R (v. 3.1.2; http://www.R-project.org/). Lastly, spatial autocorrelation among the residuals of the models was verified by Moran’s *I* correlogram (1 –predicted SDMs values for each location) [62].

### Model validations and comparisons

To assess each model’s efficiency, we compared the predicted values with the originals ones through (i) the Area Under the ROC (*Receiver Operator Characteristics*) Curve (AUC) [63, 64], (ii) TSS [60] and (iii) Boyce’s Index (BI) [64]. AUC varies from 0 (worse than a random model with the value 0.5) to 1 (perfect model), while TSS and BI varies from −1 to 1 (positive values indicate predictions consistent with the evaluation data set, 0 indicates that the model is similar to a random model). To classify the accuracy of validations, we followed Swets [65]: 0.90–1.00 = excellent; 0.80–0.90 = good; 0.70–0.80 = fair; 0.60–0.70 = poor; 0.50–0.60 = fail. We carried out ten *k-fold cross-validations* alternatively using a random sub-sample of 50% of locations to calibrate the models and the remaining 50% to validate them [66]. *Cross-validations* were carried out in R (v. 3.1.2; http://www.R-project.org/) through the packages BIOMOD2 [61] and ECOSPAT [67]. Moreover, to assess whether the nine models used provided consistent predictions in terms of variable ranking, we performed a simple linear correlation (Spearman’s test), using pair-wise comparisons for all models [68].

## Results

We removed 81 autocorrelated locations (within a distance of 1900 m) and thus we developed SDMs with a total of 103 pine marten locations. We did not find multicollinearity among the 11 predictor variables (Table 1) and thus we used all predictors for further analyses. Moreover, autocorrelation among the residuals of the nine SDMs, as well as those of the EP, were not significant and thus we considered all of them as accurate.

K-fold cross-validations showed significant values for all the evaluation methods of all distribution models (Table 2), with values ranging between 0.904 and 0.998 for AUC, 0.801 and 0.989 for TSS and from 0.802 to 0.981 for BI (Table 2). Specifically, considering both AUC and TSS statistics, RF showed the highest predictive power while MARS the lowest; considering BI, EP showed the highest predictive accuracy, while ANN the lowest (Table 2).

**Table 2:** Model evaluation of the nine species distribution methods (see the methods section for abbreviations) and their ensemble prediction (EP).

Model	AUC	TSS	BI
**ANN**	0.917 ± 0.006*	0.807 ± 0.071*	0.802 ± 0.032*
**BRT**	0.972 ± 0.027*	0.891 ± 0.043*	0.873 ± 0.041*
**CTA**	0.915 ± 0.023*	0.849 ± 0.057*	0.811 ± 0.022*
**FDA**	0.905 ± 0.025*	0.803 ± 0.002*	0.918 ± 0.014*
**GAM**	0.947 ± 0.021*	0.864 ± 0.077*	0.873 ± 0.041*
**GLM**	0.911 ± 0.086*	0.805 ± 0.011*	0.982 ± 0.017*
**MARS**	0.904 ± 0.066*	0.801 ± 0.021*	0.909 ± 0.057*
**MAXENT**	0.942 ± 0.056*	0.865 ± 0.082*	0.964 ± 0.035*
**RF**	0.998 ± 0.002*	0.989 ± 0.011*	0.804 ± 0.088*
**EP**	0.951 ± 0.048*	0.902 ± 0.022*	0.981 ± 0.019*

Area Under the Curve (AUC) ranges between 0 and 1 (worse than a random model and best discriminating model, respectively). True Skill Statistic (TSS) and Boyce’s Index (BI) ranges between −1 and 1 (higher values indicate a good predictive accuracy, while 0 indicates random prediction). Average values ± standard deviations are shown (*: P < 0.001).

**Fig 2:**
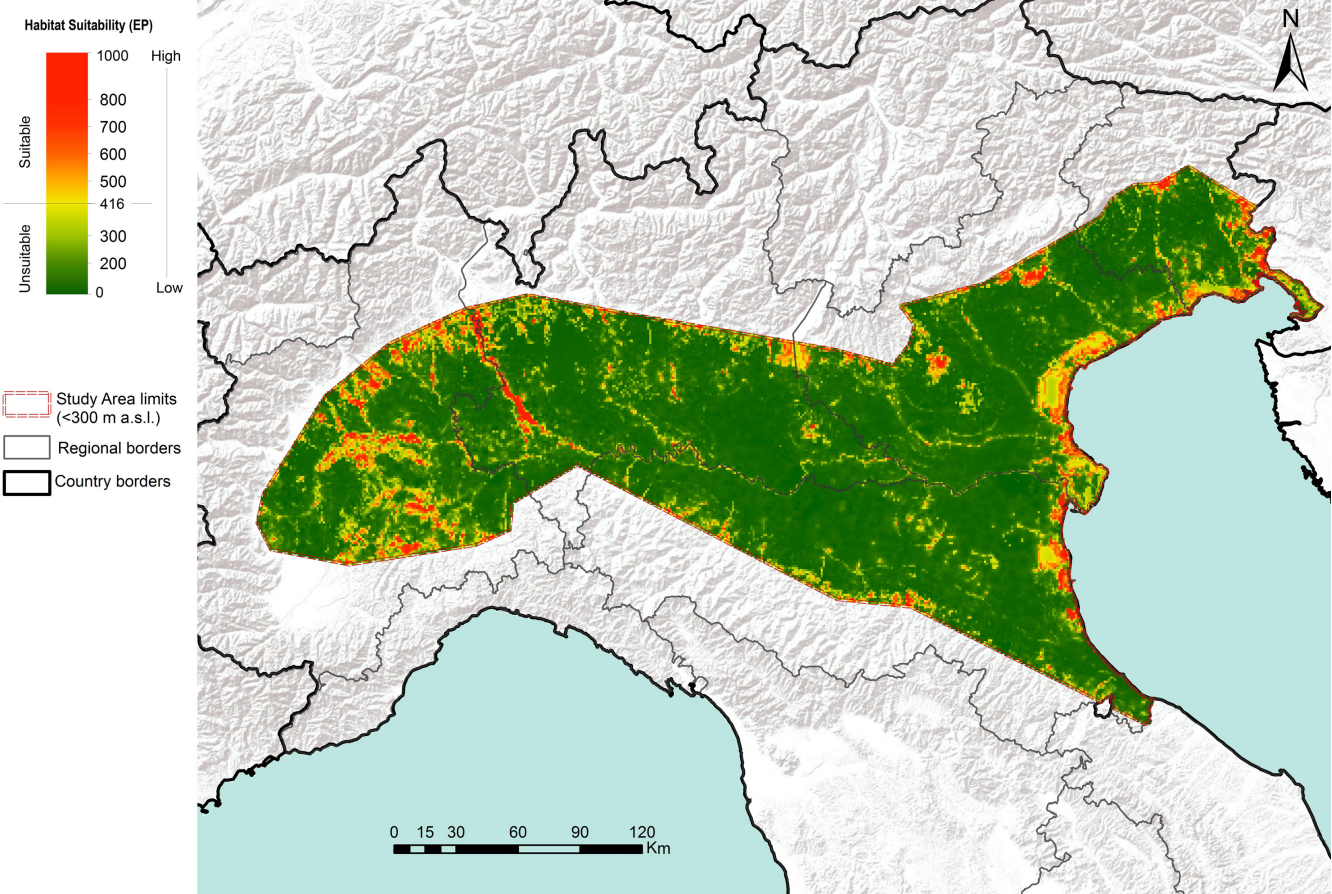
Habitat suitability map of the pine marten obtained by ensemble Species Distribution Models (green-red scale indicates lower-higher species occurrence probability).

Based on the resulting threshold value (416), EP predicted a total of 482 suitable patches for the pine marten, occupying a total of 4366 km^2^ (8.31% of the total study area; Fig 2), with the most important variables related to the species’ occurrence being the distance from watercourses (38.1% contribution), distance from woods (37.8% contribution) and, to a lesser extent, habitat diversity (11.2% contribution) and distance from roads (10.7% contribution; Table 3).

**Table 3:** Variable importance (%) ranking by the nine distribution methods (see the methods section for abbreviations) with respect to the ensemble prediction (EP).

Variables	Model									
	EP	RF	BRT	GAM	CTA	FDA	MARS	MAXENT	GLM	ANN
Grasslands	0.007	0.001	0.000	0.049	0.000	0.020	0.038	0.046	0.000	0.000
Poplar plantations	0.012	0.000	0.006	0.050	0.000	0.023	0.060	0.047	0.018	0.008
Human settlements	0.014	0.001	0.000	0.117	0.000	0.000	0.000	0.078	0.029	0.000
Woodlands	0.021	0.028	0.004	0.016	0.000	**0.535**	**0.940**	**0.449**	0.044	0.025
Cultivated fields	0.022	0.139	0.003	0.025	0.000	0.000	0.295	0.298	0.005	0.063
Distance to human settlements	0.030	0.052	0.028	0.127	0.000	0.050	0.062	0.105	0.047	**0.365**
Human population density	0.085	0.098	0.167	0.099	0.100	0.000	0.093	0.171	0.104	0.162
Distance to roads	0.107	0.096	0.060	0.178	0.135	0.020	0.032	0.141	0.032	0.232
Habitat Shannon Diversity Index	0.112	0.073	0.069	0.167	0.179	0.000	0.000	0.123	**0.257**	**0.282**
Distance to woods	**0.378**	**0.214**	**0.440**	**0.409**	**0.600**	0.000	0.000	0.267	0.073	0.223
Distance to watercourses	**0.381**	**0.446**	**0.384**	**0.291**	**0.590**	**0.378**	**0.552**	**0.517**	**0.194**	0.178

**Fig 3:**
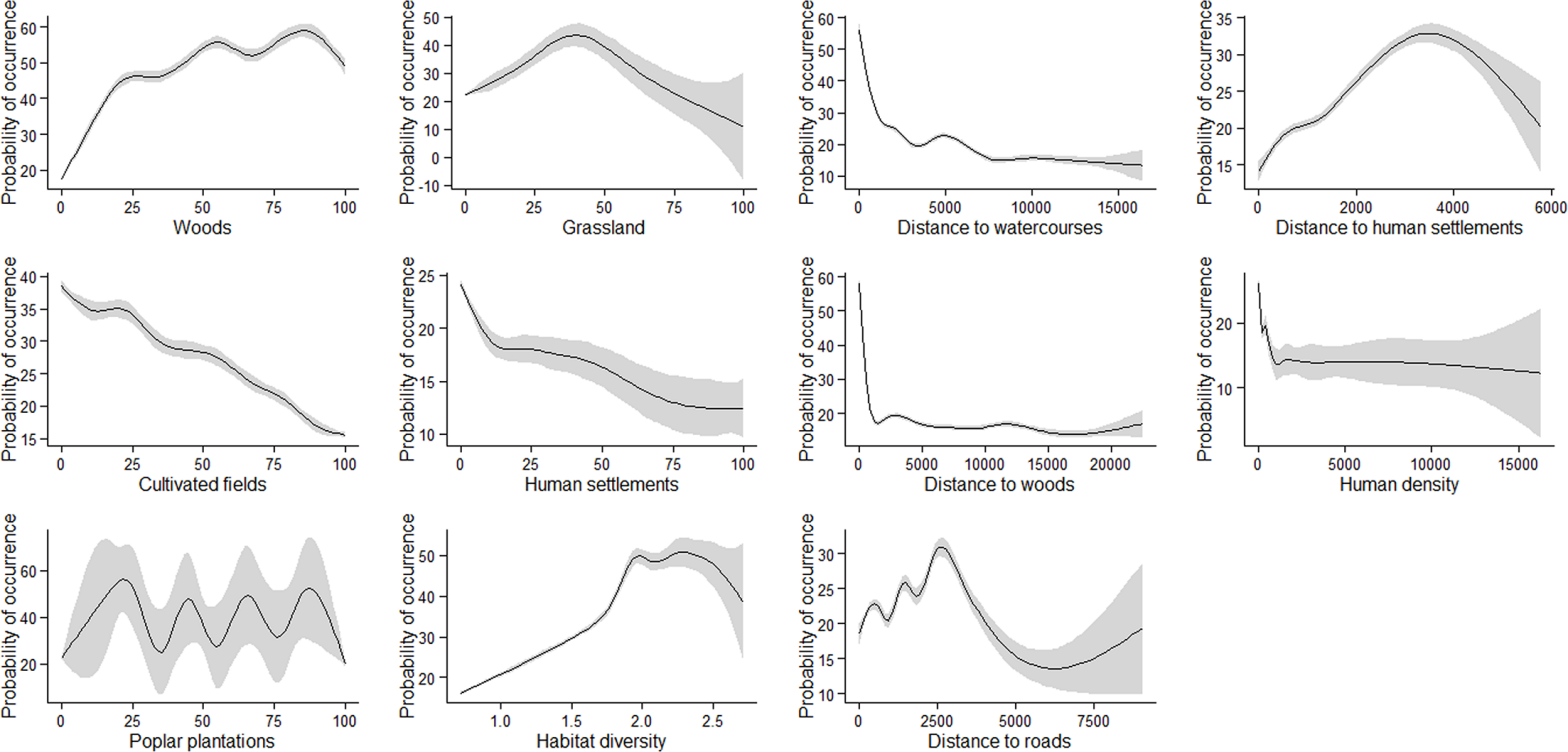
Response curves and 95% confidence intervals (in grey) of the probability of pine marten occurrence derived by the ensemble prediction of the nine species distribution models in relation to predictor variables values.

Actually, the probability of pine marten occurrence decreased as the distance from both water bodies and woods rose, while it increased with habitat diversity (Fig 3). Distance to roads showed an unimodal relationship peaking approximately at 2500 m (Fig 3). ANN was the only model which did not rank either distance from water bodies or distance from woods as the most important variables (Table 3). All models’ ranks were significantly correlated with the consensus rank (P = 0.035–0.0008), except for those provided by MARS (*ρ* = − 0.11, P = 0.75) and FDA (*ρ* = − 0.06, P = 0.87).

## Discussion

Our approach led us to identify the main environmental and anthropogenic factors affecting pine marten distribution in the western River Po plain, and outline the ecological requirements of pine martens in cultivated lowlands.

The main role played by the distance from bodies of water clearly reflects the importance of riparian corridors for marten expansion in a landscape largely dominated by crops and urban areas. Riparian zones have been reported to assist the range expansion of several mammals and support a more diverse fauna than the surrounding landscape matrix [69].

Moreover, understory vegetation in riparian forests has been associated with both prey- [70] and resting site availability [71] for American martens (*Martes americana*). Accordingly, in a previous study at a finer scale, Balestrieri et al [37], demonstrated by faecal DNA analysis that pine martens are currently widespread throughout the valley of the River Ticino, a left-hand major tributary of the River Po, where they have displaced the stone marten.

Woods are key habitat features for the pine marten, which, accordingly, has been long considered a forest-specialist [72, 73]. The pine marten is associated with mountainous forests and medium-extent agroforestry mosaics, while open and urban areas are generally avoided [74, 75].

Also in agricultural and rural areas, the abundance of pine martens has been shown to depend on the structure and degree of fragmentation of residual woods [24, 74], where resting sites are almost exclusively established [76]. Moreover in fragmented woods, martens often move along wood margins, hedgerows and corridors and tend to remain close to canopy cover [77, 78]. Therefore, open areas and highways have been reported to be the major obstacle to gene flow in lowlands [79].

In temperate regions, heterogeneous landscapes promote high biodiversity [17], and offer larger small mammal availability to predators than continuous forests [80, 81]. Wood patch borders associate cover with high prey density; therefore, below a threshold level, forest fragmentation can enhance food availability to martens and also the predator’s density [82]. For example, introduced Eastern cottontails (*Sylvilagus floridanus*) often select wood–field margins as resting sites [83], and have been reported to be a major food resource for pine martens in the study area [84].

The pattern of variation in the probability of pine marten occurrence with the distance from roads is consistent with previous results about wolves [85], and may derive from individual differences in marten response to roads [86]. In turn, this response may depend on both familiarity of the landscape, traffic intensity and road passage availability.

Although model accuracy should always be tested with independent data [87], since pine marten expansion is a recent phenomenon occurring only in the western of the River Po plain [24], a statistically independent data set for validation of our ensemble prediction was not available. We are confident that our cross-validation procedure allowed a final evaluation quasi-independent of a particular realisation of random split [32, 88]. Moreover, as cross-validation implies that variability in model accuracy is interpreted as a measure of the sensitivity of model results to the initial conditions rather than a measure of predictive accuracy [89], model averaging may improve the accuracy of projected potential distributions [90].

Although our aim was not to compare the performances of individual models, based on variable ranking and statistical evaluation ANN, FDA and MARS produced forecasts very dissimilar from the ensemble prediction, while RF performed the best. In multi-model comparisons, ANN often modelled species–environment relationships less accurately than other methods [91, 92], while RF gave the best results [34, 92]. Variation in modelling success between techniques is a common output, which further emphasizes the benefits of combining several methods [36, 93, 94].

Although, similarly to previous recent SDMs [95, 96], our model was implemented at a local (i.e. regional) scale, we argue that the environmental variable constraining pine marten presence in agricultural habitats of NW Italy can be useful to project its distribution throughout the River Po plain. Our ensemble projection suggests that the potential for pine marten expansion is high only for the western part of the River Po plain. In this area, south of the River Po the stone marten is currently the only or dominant marten species, suggesting that, flowing from west to east, the watercourse may act as a barrier to pine marten southward expansion from the Alps [16]. However, the relatively large availability of areas suitable for the pine marten allows predictions that it may colonize the whole western Po plain in the near future. In such a case, interspecific competition may result in stone marten decline, as has happened for the left-side river plain. In contrast, the negligible residual wood cover in large areas of the central and eastern plain makes them unsuitable marten habitats, although riparian corridors may allow pine marten descent from the Alps and Apennines. The potential for pine marten colonization in the pine wood patches (*Pinus pinaster* and *P*. *pinea*) of the Adriatic coast is supported by the recent southward expansion of the red squirrel (*Sciurus vulgaris*), which has probably followed the major Alpine rivers [97], and penetration of the Venetian plain by the golden jackal (*Canis aureus moreoticus*), along the valley of the River Piave [98].

In fragmented habitats, carnivores concentrate in the remnant forest patches [99, 100] and are often detected more along riparian habitats than into the surrounding agricultural matrix [101]. Accordingly, genetic data suggest that, possibly as a consequence of constraints on dispersal imposed by the surrounding open habitats, pine marten density in lowland riparian woods of NW Italy is among the highest ever recorded throughout its European range [20]. Our results demonstrate that pine marten occurrence in intensively cultivated areas strictly depends on the preservation of existing forest patches and suggest that conservation management should seek to enhance the functional connectivity provided by riparian corridors.

## Supporting Information

S1 Table Coordinates of pine marten records in the western valley of the River Po (Italy). (DOCX)

S1 Text Permission from ESRI to publish Fig 1 under the specific Creative Commons Attribution License (CCAL), CC BY 4.0. (PDF)

S2 Text Permission from the European Environment Agency (EEA) to publish Fig 1 under the specific Creative Commons Attribution License (CCAL), CC BY 4.0. (PDF)
