# Supplementary material for: Modelling the Distribution of Forest-Dependent Species in Human-Dominated Landscapes: Patterns for the Pine Marten in Intensively Cultivated Lowlands
Source: PLoS One. 2016 Jul 1;11(7):e0158203. doi: 10.1371/journal.pone.0158203 (PMC4930197; doi:10.1371/journal.pone.0158203)
Supplement: S1 Table — (DOCX) [file pone.0158203.s001.docx]

**S1 Table. Coordinates of pine marten records.**

| **x** | **y** | **Type** |  | **x** | **y** | **Type** |
| --- | --- | --- | --- | --- | --- | --- |
| 499666 | 5009151 | cameratrap |  | 500003 | 5010102 | DNA |
| 498527 | 5009552 | cameratrap |  | 500288 | 5010550 | DNA |
| 499256 | 5009801 | cameratrap |  | 500204 | 5010699 | DNA |
| 499600 | 5009827 | cameratrap |  | 499984 | 5011218 | DNA |
| 498227 | 5011090 | cameratrap |  | 497185 | 5010619 | DNA |
| 496368 | 5011318 | cameratrap |  | 498369 | 5010969 | DNA |
| 497665 | 5011912 | cameratrap |  | 498518 | 5011037 | DNA |
| 496659 | 5012318 | cameratrap |  | 498336 | 5011049 | DNA |
| 496352 | 5013377 | cameratrap |  | 498088 | 5011439 | DNA |
| 495865 | 5013843 | cameratrap |  | 497780 | 5011605 | DNA |
| 496507 | 5013936 | cameratrap |  | 498206 | 5011651 | DNA |
| 494916 | 5016034 | cameratrap |  | 498770 | 5013414 | DNA |
| 435199 | 5046782 | cameratrap |  | 499110 | 5013467 | DNA |
| 496667 | 5012114 | DNA |  | 498953 | 5013539 | DNA |
| 496549 | 5012409 | DNA |  | 495345 | 5014366 | DNA |
| 496474 | 5012434 | DNA |  | 495067 | 5014595 | DNA |
| 498721 | 5004554 | DNA |  | 495133 | 5014631 | DNA |
| 498333 | 5005101 | DNA |  | 495501 | 5014889 | DNA |
| 500298 | 5009375 | DNA |  | 495374 | 5015089 | DNA |
| 495082 | 5010157 | DNA |  | 496339 | 5015162 | DNA |
| 495621 | 5010753 | DNA |  | 493862 | 5017387 | DNA |
| 495425 | 5011429 | DNA |  | 500546 | 5009210 | DNA |
| 494988 | 5012070 | DNA |  | 500351 | 5009315 | DNA |
| 495027 | 5012092 | DNA |  | 498789 | 5009353 | DNA |
| 494376 | 5013957 | DNA |  | 500247 | 5009448 | DNA |
| 495012 | 5007574 | DNA |  | 500024 | 5009766 | DNA |
| 497220 | 5007734 | DNA |  | 498238 | 5010203 | DNA |
| 495769 | 5010836 | DNA |  | 497120 | 5010739 | DNA |
| 498618 | 5011269 | DNA |  | 497077 | 5010771 | DNA |
| 498606 | 5011285 | DNA |  | 496801 | 5010859 | DNA |
| 498625 | 5011415 | DNA |  | 496781 | 5011027 | DNA |
| 495048 | 5011488 | DNA |  | 495103 | 5011213 | DNA |
| 496670 | 5011594 | DNA |  | 498080 | 5011264 | DNA |
| 496853 | 5011790 | DNA |  | 494760 | 5011723 | DNA |
| 497946 | 5012270 | DNA |  | 494886 | 5011749 | DNA |
| 497865 | 5012944 | DNA |  | 496795 | 5012055 | DNA |
| 495351 | 5015144 | DNA |  | 494932 | 5013661 | DNA |
| 494618 | 5017064 | DNA |  | 494110 | 5014010 | DNA |
| 499287 | 5005213 | DNA |  | 496268 | 5015303 | DNA |
| 499178 | 5005645 | DNA |  | 491972 | 5020343 | DNA |
| 499977 | 5008986 | DNA |  | 470332 | 4988402 | DNA |
| 498796 | 5009304 | DNA |  | 495406 | 5015053 | DNA |
| 498764 | 5009389 | DNA |  | 494943 | 5015751 | DNA |
| 500204 | 5009492 | DNA |  | 494780 | 5016116 | DNA |
| 500068 | 5009700 | DNA |  | 494884 | 5016788 | DNA |
| 499990 | 5009869 | DNA |  | 494075 | 5016879 | DNA |
| **x** | **y** | **Type** |  | **x** | **y** | **Type** |
| 497344 | 5003858 | DNA |  | 455608 | 5051769 | roadkill |
| 497264 | 5004348 | DNA |  | 390311 | 4976976 | roadkill |
| 497320 | 5004572 | DNA |  | 451430 | 5029360 | roadkill |
| 497447 | 5004737 | DNA |  | 499707 | 5004007 | roadkill |
| 497406 | 5004855 | DNA |  | 436352 | 5043024 | roadkill |
| 497278 | 5004982 | DNA |  | 406674 | 5012125 | roadkill |
| 501745 | 5008973 | DNA |  | 437535 | 5044640 | roadkill |
| 440199 | 5039422 | DNA |  | 484250 | 5032800 | roadkill |
| 437655 | 5043083 | DNA |  | 445192 | 5049939 | roadkill |
| 432719 | 5043548 | DNA |  | 498521 | 5011065 | roadkill |
| 434176 | 5043898 | DNA |  | 422784 | 4958526 | roadkill |
| 490200 | 5021183 | DNA |  | 430570 | 5032553 | roadkill |
| 490536 | 5021810 | DNA |  | 441748 | 5038334 | roadkill |
| 452326 | 5030663 | DNA |  | 430346 | 5033852 | roadkill |
| 437662 | 5038375 | DNA |  | 495609 | 5006774 | roadkill |
| 435606 | 5039135 | DNA |  | 498948 | 5013899 | roadkill |
| 485917 | 5032369 | DNA |  | 446606 | 5019928 | roadkill |
| 485876 | 5032502 | DNA |  | 439203 | 5044562 | roadkill |
| 477019 | 5041696 | DNA |  | 438178 | 5046115 | roadkill |
| 453207 | 5026745 | DNA |  | 448146 | 5050932 | roadkill |
| 453265 | 5026881 | DNA |  | 441769 | 5038303 | roadkill |
| 477502 | 5046022 | DNA |  | 439392 | 5046134 | roadkill |
| 425997 | 5025512 | DNA |  | 483804 | 5035696 | roadkill |
| 452944 | 5036430 | DNA |  | 395347 | 4968198 | roadkill |
| 453122 | 5039946 | DNA |  | 393743 | 4965145 | roadkill |
| 448121 | 5044170 | DNA |  | 407507 | 4972559 | roadkill |
| 448272 | 5044277 | DNA |  | 430546 | 5032275 | roadkill |
| 441926 | 5007787 | DNA |  | 443431 | 5042084 | roadkill |
| 447593 | 5029177 | DNA |  | 442909 | 5044342 | roadkill |
| 452282 | 5030615 | DNA |  | 503555 | 5008068 | roadkill |
| 441098 | 5036665 | DNA |  | 471837 | 4988968 | roadkill |
| 471572 | 5025810 | DNA |  | 376730 | 4952897 | roadkill |
| 486747 | 5025988 | DNA |  | 394296 | 4960730 | roadkill |
| 486958 | 5026234 | DNA |  | 386669 | 4980976 | roadkill |
| 499981 | 5009918 | DNA |  | 471719 | 4988944 | roadkill |
| 501691 | 5008990 | DNA |  | 499681 | 5004042 | roadkill |
| 452964 | 5036449 | DNA |  | 498827 | 5013473 | roadkill |
| 444124 | 5031042 | roadkill |  | 467855 | 5035556 | roadkill |
| 581469 | 4973458 | roadkill |  | 501303 | 5009454 | roadkill |
| 476893 | 5040143 | roadkill |  | 386429 | 4960249 | roadkill |
| 444239 | 5032880 | roadkill |  | 378876 | 4975810 | roadkill |
| 472210 | 4989107 | roadkill |  | 385689 | 4969196 | roadkill |
| 454823 | 5044679 | roadkill |  | 486608 | 5031261 | roadkill |
| 444995 | 5047679 | roadkill |  | 497725 | 5011954 | roadkill |
| 472210 | 4989107 | roadkill |  | 385408 | 4969186 | roadkill |
| 456130 | 5052004 | roadkill |  | 500058 | 5006529 | roadkill |
