# Supplementary material for: Modelling the Distribution of Forest-Dependent Species in Human-Dominated Landscapes: Patterns for the Pine Marten in Intensively Cultivated Lowlands
Source: PLoS One. 2016 Jul 1;11(7):e0158203. doi: 10.1371/journal.pone.0158203 (PMC4930197; doi:10.1371/journal.pone.0158203)
Supplement: S1 Text — (PDF) [file pone.0158203.s002.pdf]

Don Alfonso Rubio Barroso, con D.N.I. nº 02.188.551-D, en calidad de apoderado de la empresa ESRI ESPAÑA SOLUCIONES GEOESPACIALES, S.L. con domicilio en Madrid, calle Emilio Muñoz, 35 - 4ª Planta, 28037 y con C.I.F. nº B86900057.

AUTORIZA A:

Aritz Ruiz, doctor en el Departamento de Didáctica de la Matemática y de las Ciencias Experimentales de la UPV/EHU, a poder publicar en la revista científica PLOS One, unas figuras o representaciones cartográficas creadas a partir de mapa base o basemap (concretamente el World Topographic) propiedad de Esri.

Y para que conste a los efectos oportunos firmo la presente en Madrid, a 26 de abril de 2016.

Fdo.: Alfonso Rubio Barroso

Consejero Delegado

Esri España Soluciones Geoespaciales, S.L.
Emilio Muñoz, 35
28037 Madrid, España
C.I.F.: B-86900057

T +34 91 559 43 75
F +34 91 010 86 65
informacion@esri.es

esri.es

Inscrita en el Registro Mercantil de la Provincia de Madrid Hoja nº M-573.864, Folio 25, Tomo 31.887 Madrid
