# Supplementary material for: Modelling the Distribution of Forest-Dependent Species in Human-Dominated Landscapes: Patterns for the Pine Marten in Intensively Cultivated Lowlands
Source: PLoS One. 2016 Jul 1;11(7):e0158203. doi: 10.1371/journal.pone.0158203 (PMC4930197; doi:10.1371/journal.pone.0158203)
Supplement: S2 Text — (PDF) [file pone.0158203.s003.pdf]

From: **copyrights** <copyrights@eea.europa.eu>
Date: 2016-04-13 11:48 GMT+02:00
Subject: RE: re-use of content on the EEA website -Corine landcoverdata
To: Aritz Ruiz <aritz.ruiz@ehu.eus>

Dear Mr Ruiz-González

Re-use of content on the EEA website which is the property of the European Environment Agency (EEA) and for which the EEA holds the rights of use, is permitted free of charge for commercial or non-commercial purposes, provided that the source is acknowledged and that the entire item is reproduced.

The EEA re-use policy follows Directive 2003/98/EC of the European Parliament and the Council on the re-use of public sector information throughout the European Union and the Commission Decision of 12 December 2011 on re-use of Commission documents. As spelled out in article 6 in the latter, *“Documents shall be made available for reuse without application unless otherwise specified”*.

You thus need no advance permission to re-use datasets from the Corine Land Cover data found on our website.

With kind regards

Ove Caspersen

Ove Caspersen

Project manager

Marketing, exhibitions and licencing

Kongens Nytorv 6

1050 Copenhagen K, Denmark

eea.europa.eu

Phone:+45 33367100/29612803

Fax:+45 33367199
